# Paintable and writable electrodes using black conductive ink on traditional Korean paper (Hanji)†

**DOI:** 10.1039/d0ra04412a

**Published:** 2020-06-29

**Authors:** Yong Jun Kim, Sunyoung Yoon, Yong-Hwan Cho, Gyewon Kim, Han-Ki Kim

**Affiliations:** School of Advanced Materials Science and Engineering, Sungkyunkwan University Suwon Gyeonggi-do 16419 Korea; Department of Fine Arts, Sungkyunkwan University 25-2, Sungkyunkwan-ro, Jongno-gu Seoul Republic of Korea hankikim@skku.edu +82-31-201-2462 +82-31-205-2462

## Abstract

We demonstrate black conductive ink (BCI) that is writable and paintable on traditional handmade Korean paper (Hanji) for application as a high performing electrode. By optimal mixing of Ag nanowire (Ag NW) suspension and poly(3,4-ethylenedioxythiophene):poly(styrenesulfonate)(PEDOT:PSS) solution in standard charcoal-based blank ink, we synthesized BCI suitable for writing and painting on Hanji with a normal paintbrush. Due to the shear stress induced by the paintbrush bristles, the Ag NW and PEDOT:PSS mixture was uniformly coated on the porous cellulose structure of Hanji and showed a low sheet resistance of 11.7 Ohm per square even after repeated brush strokes. Moreover, the brush-painted electrodes on Hanji showed a constant resistance during tests of inner/outer bending and folding due to the outstanding flexibility of the Ag NW and PEDOT:PSS mixture that filled the porous cellulose structure of Hanji. Therefore, the pictures drawn in the BCI on Hanji exhibited a level of flexibility and conductivity sufficiently high to enable the BCI to function as an effective electrode even when the paper substrate is wrinkled or crumpled. The successful operation of the paintable interconnector and heater on Hanji indicates the high potential of the brush-painted electrodes that can be used in various social and cultural fields, including fine art, fashion, interior design, architecture, and heating industry.

## Abstract 2

Brush painted black conductive ink on traditional Korean paper “Hanji” having durability, good ventilation, and air permeability, and being waterproof was demonstrated in paintable interconnectors and heaters.

## Graphical abstract

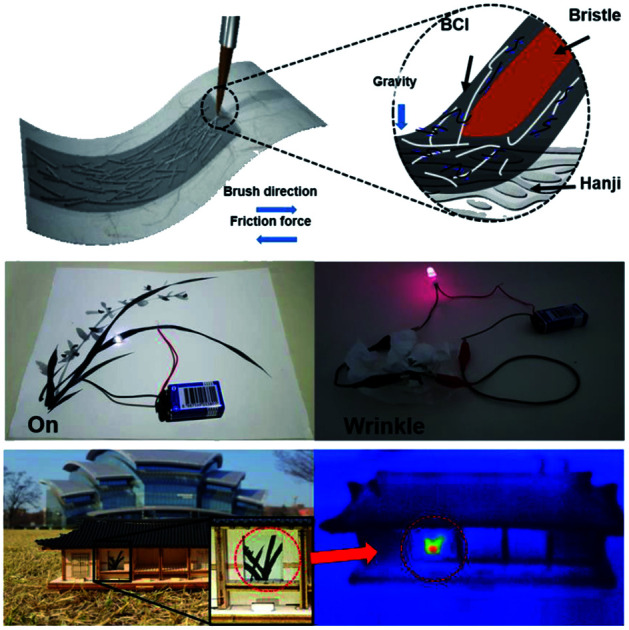

## 1 Introduction

Paper electronics continue to be investigated due to the various advantages of a paper substrate such as its renewability, light weight, low cost, deformability, and flexibility.^1–4^ Since paper also offers electrical insulation like a glass or polymer substrate, it can be used as an insulating layer or a substrate for electronic devices such as transistors,^5,6^ heaters,^2,7,8^ energy storage devices,^9–13^ actuators,^6^ and sensors.^4,14^ Among different types of paper substrate, Hanji, a handmade traditional Korean paper consisting of multiple laminated sheets, can be used as a simple and cost-effective substrate for paper electronics. Due to the various advantages of Hanji, such as durability, good ventilation, air permeability, and being waterproof, this traditional Korean paper has been used as the main substrate for oriental art and the surface material covering for the wooden door frames of traditional Korean houses.^15^ In oriental art, a paint brush has been a main tool for drawing pictures or writing characters on Hanji.^16^ Recently, the brush painting of oriental art has been applied to the solution-based fabrication of optoelectronic devices as a simple coating method due to its various advantages such as cost-efficiency, simplicity, and ambient coating process.^17–20^ The solution-processed layer within organic solar cells, strain sensors, thin film transistors and perovskite solar cells has been fabricated *via* simple brush painting on rigid or flexible substrates.^19–22^ In the fabrication of solution-processed devices using brush painting, the film thickness could be controlled from ∼10 nm up to a few hundred nanometers, which has facilitated its application to different kinds of brush-paintable device.^20,23^ As brush painting is a human-oriented coating technology, users can configure electrodes of various shapes without any limit of form or design. Our group has previously reported brush paintable electrodes made of Ag nanowires (Ag NWs),^17,24,25^ carbon nanotubes(CNTs),^26^ poly(3,4-ethylenedioxythiophene):poly(styre-nesulfonate)(PEDOT:PSS),^25^ Sn-doped In_2_O_3_ nanoparticles(NPs),^21^ and Ti-doped In_2_O_3_ NPs^18^ on glass substrate for use as transparent electrodes for organic solar cells. Although brush painting has been applied for the coating process of solution ink, most of the cases adopted rigid glass as a substrate. Recently our group demonstrated brush-painted electrode mixture consisting of Ag NWs and PEDOT:PSS on stretchable polyurethane (PU) substrate for stretchable and wearable electronics.^17^ We successfully coated the transparent, flexible, and stretchable electrode with a sheet resistance of 7.5 Ohm per square and transmittance of 88.64% on a PU substrate *via* a simple brush-painting process.^20^ Heo *et al.* has reported a yarn-type supercapacitor using twistable Hanji, coated with reduced graphene oxide and single walled CNT.^9^ Go *et al.* has investigated Ag NW and ZnO hybrid electrode and reported that Hanji is a promising substrate to overcome the limit of conventional paper as a soft electrode due to its higher mechanical resistance and excellent chemical resistance.^2^ Seo *et al.*, has demonstrated a dry transfer process to transfer Ag NPs and NW composite from PET to Hanji substrate.^13^ They fabricated the Hanji-based composite current collector for application to flexible supercapacitors. Although brush painting and Hanji substrate have been reported to be a simple and cost-effective coating method and promising paper substrate, no study in the literature has reported brush painting on the Hanji substrate for paper electronics and solid research focused on brush painting on flexible Hanji substrate remains lacking.

Herein, to fill this research gap, we describe the synthesis and optimization of black conductive ink (BCI) for brush painting on Hanji substrate to realize paper interconnectors and paper heaters. The BCI was synthesized from Ag NW suspension, PEDOT:PSS solution, and charcoal NPs ink and directly painted on Hanji at various mixing ratios of Ag NW suspension and PEDOT:PSS solution. We investigated in depth the electrical and mechanical properties of the brush-painted electrodes on Hanji. In particular, we demonstrated BCI drawing and heater on Hanji through the process of brush painting. Due to their high conductivity, brush-painted electrodes on Hanji could act as interconnectors for commercial LEDs. Furthermore, we successfully verified the operation of brush paintable interconnectors on Hanji and canvas, both of which can illuminate commercial LEDs.

## 2 Experimental

### 2.1 Synthesis of black conductive ink (BCI)

**Fig. 1:**
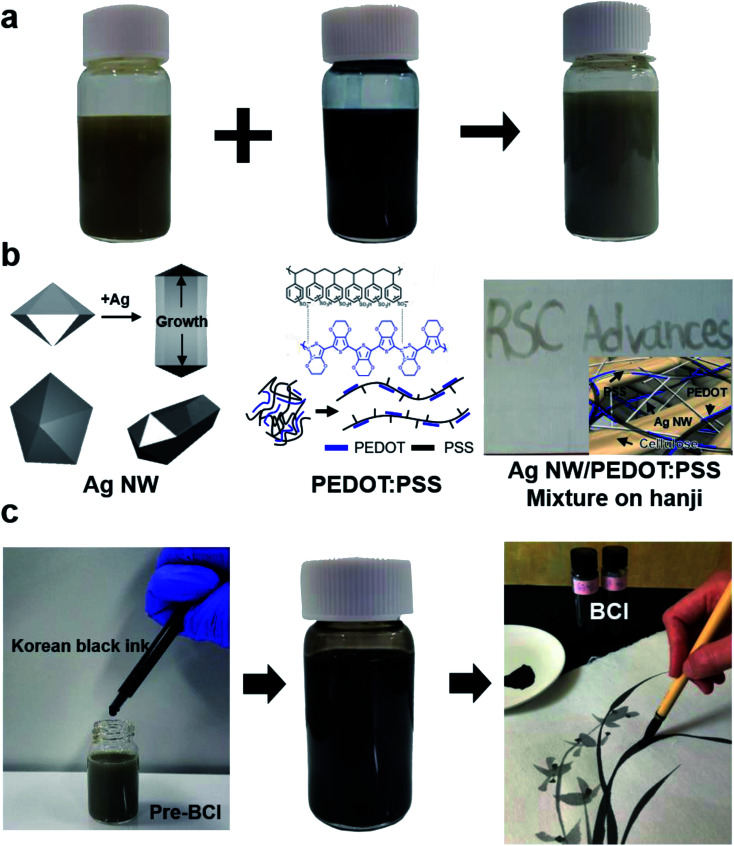
(a) A schematic diagram of the fabrication of pre-BCI ink. Ag NW suspension and PEDOT:PSS solution were mixed as a function of volume ratio. (b) In pre-BCI ink, Ag NW acts as a main conduction path and PEDOT:PSS as a conducting matrix connecting Ag NWs. Before mixing into the charcoal-based black ink, the pre-BCI ink showed a gray color. The brush-painted RSC Advances showed shallow gray color. (c) Synthesis of BCI by mixing common Korean black ink and pre-BCI ink. The right-side picture shows the black color of the painted orchid on Hanji using optimal BCI.

The BCI was synthesized by mixing 0.3 wt% DI based Ag NW suspension(20–30 nm diameter and 20–30 μm length), (SNWI-006a,FLEXIO Co., Ltd), and 1.2 wt% DI based PEDOT:PSS (weight ratio of 1 : 2.5) solution (Clevios PH1000, Heraeus) in a common charcoal-based black Korean ink (Meok). First, we fabricated mixtures (pre-BCI) of Ag NWs and PEDOT:PSS solution with various mixing volume ratios (20 ml : 1 ml, 20 ml : 3 ml, 20 ml : 5 ml, and 20 ml : 10 ml) to determine the optimal mixing ratio. We then fabricated the BCI by adding common black ink to optimal pre-BCI at a mixing volume ratio of 1 ml : 9 ml, as shown in Fig. 1(a). The BCI was stirred on a magnetic stirrer for three hours to ensure uniform mixing.

### 2.2 Brush painting in black conductive ink (BCI) on Hanji paper

The BCI electrode made at the optimal mixing condition was coated on a typical Hanji paper using a Nylon fibril paintbrush with a width of 1.5 cm, which is used for writing characters and painting pictures. We also coated the BCI on a typical canvas (ROYAL 2580 KORF) to show its applicability to western-style paintings. The BCI electrode painted on Hanji and canvas can also function as visual art itself. The pictures and characters, after being drawn in the BCI by the paintbrush, were dried in a convection oven at 35 °C for 10 minutes. The electrical properties of the brush-painted electrodes were analyzed with a four-point probe (FPP-HS8, DASOL ENG). The surface morphology of bare and BCI-coated Hanji was analyzed using field emission scanning electron microscopy (JSM-7600F, JEOL). The mechanical flexibility of the brush-painted electrodes on the Hanji substrate was investigated by our own lab-designed inner/outer bending test and folding test systems. To confirm the mechanical stability of the brush-painted electrodes on Hanji, we subjected them to repeated bending cycling and folding tests up to 1000 cycles.

### 2.3 Fabrication of brush paintable interconnectors and heaters

To show promising applications of the brush-painted electrodes on Hanji, general light emitting diodes(LEDs) were embedded on the painting, which acted as interconnectors. To confirm the conductivity of the brush-painted picture, DC power was applied to the LEDs, and transmitted through the lines and forms of brush painting. In addition, brush painting in BCI was applicable for paintable heaters. To fabricate the paper-based heater, a copper tape was connected to brush painting on Hanji as contacting electrode at the end of both sides. A DC voltage was supplied by a power supply (OPS 3010, ODA technologies) to the painted picture using a copper tape contact electrode. The temperature and thermal stability of the paper-based heater were investigated using a thermocouple and an infrared thermal imager (A35sc, FLIR).

## 3 Results and discussion

The schematic diagram in Fig. 1(a) shows the fabrication process of adding the Ag NW suspension and PEDOT:PSS solution mixing ink (Pre-BCI) into standard charcoal-based black ink as conductive elements. In BCI brush painting, the Ag NW provides a conduction pathway due to the low resistivity of the Ag NW and percolating network structure.^23,27–31^ The PEDOT:PSS chains act as a conductive matrix connecting the Ag NW network, as shown in Fig. 1(b) of right inset picture. The color of pre-BCI ink was gray, as shown in Fig. 1(a). However, when it was applied on Hanji using a paintbrush, the ‘RSC Advances’ character showed shallow gray color and semi-transparency as shown in Fig. 1(b). Therefore, pre-BCI ink was not appropriate for use in brush painting. As shown in Fig. 1(c), the addition of standard charcoal-based black ink into pre-BCI enabled the fabrication of BCI suitable for brush painting on Hanji. At the optimal volume ratio (1 ml : 9 ml) of usual black ink and pre-BCI, we were also able to fabricate a BCI for brush painting. The right-side in Fig. 1(c) shows the inner structure of the brush-painted orchid on Hanji using optimal BCI. Unlike the pre-BCI, the optimal BCI showed a typical black color. The chroma in the brush-painted oriental picture could be controlled by adding water since it is the main solvent in the BCI.

**Fig. 2:**
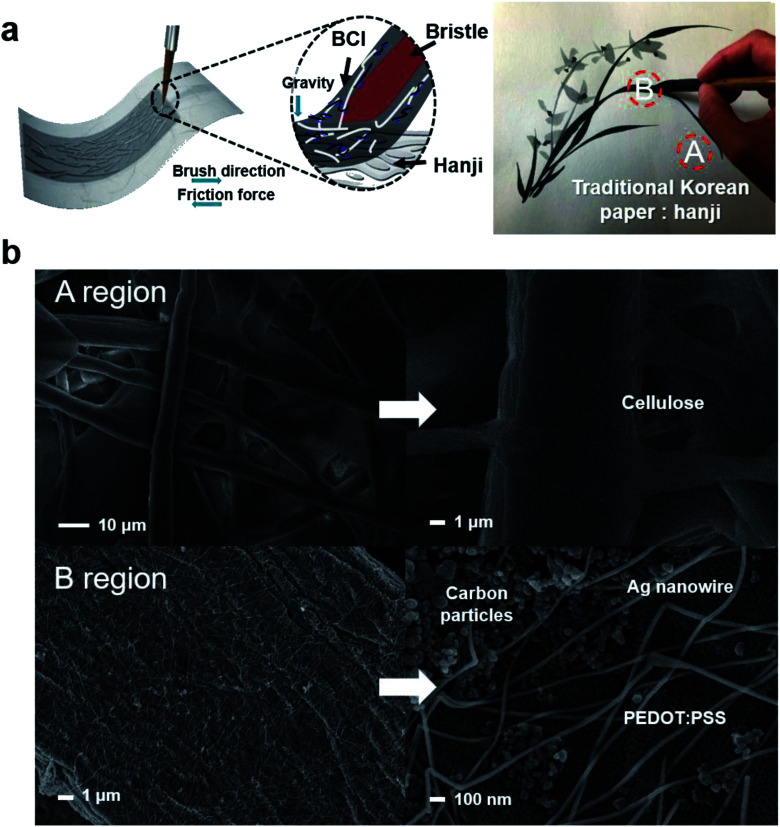
(a) Schematic illustration of the brush-painting process using BCI on Hanji for an orchid. (b) Comparative images of FESEM obtained from a pristine Hanji (A) and BCI-coated Hanji (B) by using the brush painting of orchid. The enlarged image shows the surface of cellulose fiber covered with Ag NWs and carbon nanoparticles after applying BCI onto Hanji with a paintbrush.

Fig. 2(a) shows a schematic diagram of the brush-painting process on a Hanji substrate using BCI. When a paintbrush was dipped into BCI, it absorbed a large amount of the ink. As shown in Fig. 2(a), the BCI absorbed by the paintbrush bristles was coated on the Hanji substrate at the artist's disposal. Due to the porous cellulose structure of the Hanji and shear stress caused by the bristles, BCI is easily absorbed into the Hanji.^32^ As we have discussed in our previous works, the interaction of the shear stress and friction force evenly induces the formation of a well-connected Ag NW network on the cellulose of the Hanji.^17,20,25,33^ After drying the orchid painting drawing in BCI, the surface morphology and textural properties of region A (pristine Hanji) and region B (BCI-coated Hanji) were investigated by FE-SEM, as shown in Fig. 2(b). We confirmed that a size of carbon particles is about 50–100 nm based on SEM image. The pristine Hanji region (A) showed the typical cellulose fiber structure of paper, which is indicative of a well-connected network with good entanglement. As reported by Heo *et al.*, the Hanji substrate showed a highly porous structure.^9^ However, the FESEM images revealed a different surface morphology for the BCI-coated Hanji. The cellulose fiber was well covered by percolating Ag NWs and carbon NPs after BCI brush painting. The magnified image showed that the Ag NWs formed an effective network capable of providing a current conduction path on the surface of the fiber. The Ag NW also adhered well to the fiber. The agglomerated NPs indicated carbon NPs in the BCI applicable for black color paintings.

**Fig. 3:**
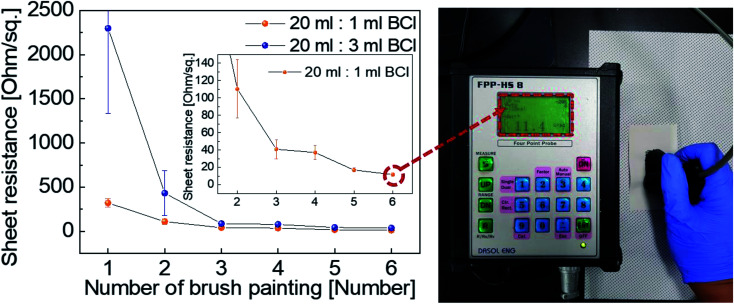
Sheet resistance of brush-painted electrode on Hanji substrate with different mixing ratios of Ag NW suspension and PEDOT:PSS solution (20 ml : 1 ml and 20 ml : 3 ml) as a function of brush stroke number. The right side picture displays the *in situ* measured sheet resistance of the brush-painted electrodes on Hanji.

Fig. 3 shows the sheet resistance of the BCI electrodes painted on a Hanji substrate as a function of the number of brush strokes. The BCI with different mixing ratios of Ag NW suspension and PEDOT:PSS solution (20 ml : 1 ml and 20 ml : 3 ml) was employed in brush painting on the Hanji substrate. Although 20 ml : 5 ml and 20 ml : 10 ml BCI were also prepared, they exhibited fairly high sheet resistance, as shown in Fig. S1.† At the first brush stroke, both electrodes showed a high sheet resistance of 321 Ohm per square (20 ml : 1 ml) and 2296 Ohm per square (20 ml : 3 ml). However, as the number of brush strokes increased, the sheet resistance of the Hanji-made electrodes decreased. After the application of six brush strokes to the Hanji surface, both electrodes displayed significantly reduced sheet resistance of 11.7 (20 ml : 1 ml) and 37.9 (20 ml : 3 ml) Ohm per square. Compared to the brush-painted electrode fabricated by BCI (20 ml : 3 ml), that by BCI (20 ml : 1 ml) showed lower sheet resistance due to its high density of Ag NWs. The decreasing sheet resistance was attributed to the effective coverage of Ag NW on the fiber and filling of the porous fiber structure with the BCI. Fig. S2† shows the XRD data of the electrodes with six brush strokes, fabricated by 20 ml : 1 ml and 20 ml : 3 ml BCI solution. Compared to the pristine Hanji, both painted electrodes showed additional (111) peaks, which were observed at 2*θ* = 38.25° and 2*θ* = 38.23°.^35,36^ Since the end of Ag NW coated on fiber was terminated by the (111) plane, the XRD plot showed a strong (111) peak. Furthermore, we investigated the electrical properties of red, green, and blue dye-added conductive ink by applying them with a paintbrush on a standard drawing paper, as shown in Fig. S3 and S4.† Like the BCI (20 ml : 1 ml and 20 ml : 3 ml) solution, the sheet resistance of the brush-painted color electrodes decreased with increasing number of brush strokes. However, the sheet resistance of the blue color electrode painted by the blue-dye conductive ink was not measured.

**Fig. 4:**
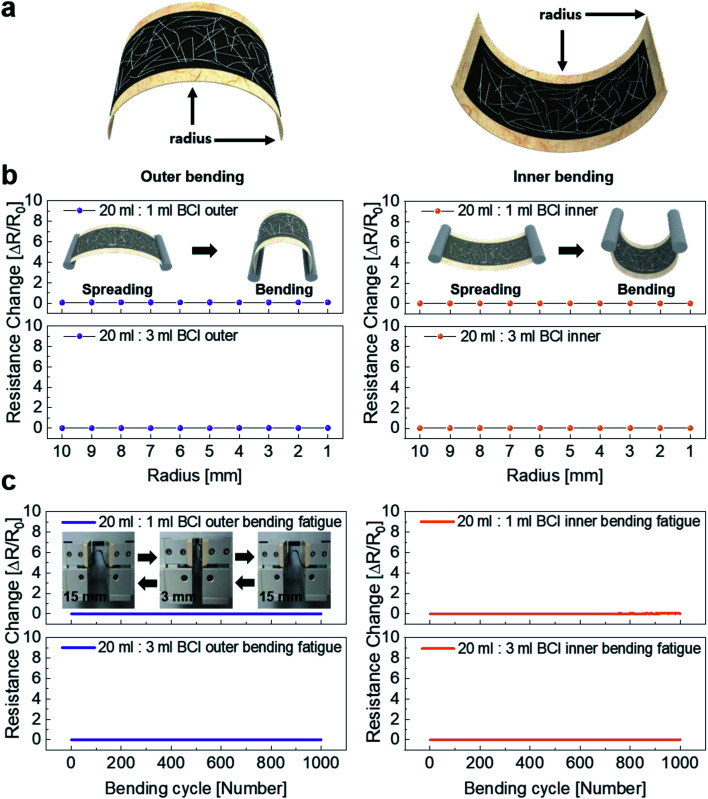
(a) Outer and inner bending of the brush-painted electrodes on Hanji experience tensile and compressive stresses. (b) The results of outer and inner bending tests of brush-pained electrodes (20 ml : 1 ml and 20 ml : 3 ml) over the decreasing bending radius range of 10 mm to 1 mm. (c) Resistance change in the dynamic bending fatigue test of the brush-painted electrodes for 1000 cycles. The inset shows a picture of the dynamic fatigue steps.

To investigate the mechanical properties of the brush-painted electrode on Hanji, we measured its resistance as a function of bending radius. Fig. 4(a) shows the definition of an inner and an outer bending radius for the bending test of the brush-painted electrodes. The brush-painted electrode on Hanji was convex and experienced tensile stress at the center of the sample for outer bending but was concave and experienced compressive stress at the center of the sample for inner bending. The mechanical properties of the brush-painted electrodes on Hanji were evaluated based on the change in resistance (*R*_change_) defined in the following eqn (1).^20,34,35^1
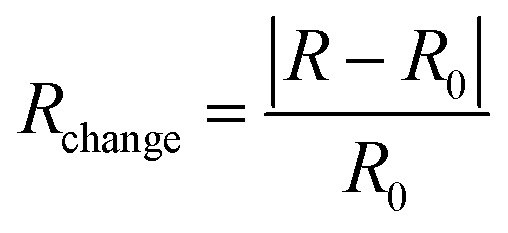
where *R* and *R*_0_ are the *in situ* measured resistance and initial resistance of the brush-electrode on Hanji, respectively. Using a lab-designed bending test system, the *R*_change_ of the brush-painted electrode was *in situ* measured as a function of outer and inner bending radius, as shown in Fig. 4(b). Regardless of an inner and outer radius mode and mixing ratio, the brush-painted electrode showed a constant resistance, which indicated the outstanding flexibility of the BCI-coated Hanji. Until a small bending radius of 1 mm, the brush-painted electrode on Hanji maintained an identical resistance with 10 mm bent samples due to the flexibility of the Hanji substrate and Ag NW network. Fig. 4(c) shows the repeated outer and inner bending fatigue test at a fixed bending radius of 3 mm. In this dynamic outer and inner fatigue test, the brush-painted electrode on Hanji showed no resistance changes for 1000 cycles.

**Fig. 5:**
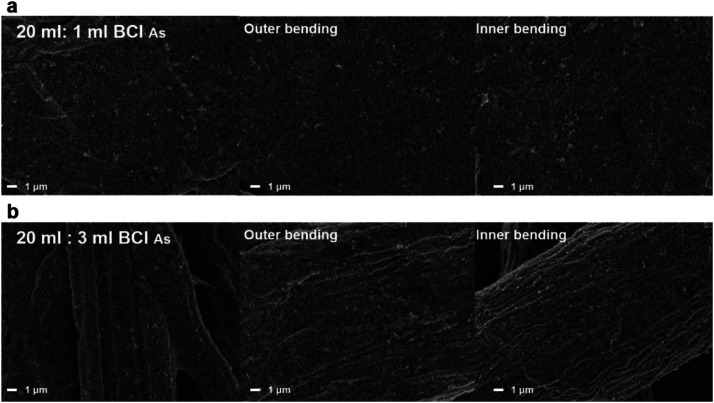
Surface FESEM images of the brush-painted electrode before and after outer/inner bending tests at a radius of 1 mm. The BCI-coated Hanji with an Ag NW suspension and PEDOT:PSS solution mixing ratio of (a) 20 ml : 1 ml and (b) 20 ml : 3 ml, respectively.

Fig. 5 shows surface FESEM images obtained from the brush-painted electrode on Hanji, with two different mixing ratios (20 ml : 1 ml and 20 ml : 3 ml) of BCI, before and after the outer and inner bending tests. Despite the outer and inner bending tests at 1 mm, the brush-painted electrode showed identical surface FESEM images to those of the as-printed electrode. Due to the durability of the cellulose fiber structure and the flexibility of the Ag NW network, the brush-painted electrode showed outstanding mechanical flexibility, regardless of the bending modes. Even after 1 mm outer and inner bending, the Ag NW network was well covered on the cellulous fiber with no delamination.

**Fig. 6:**
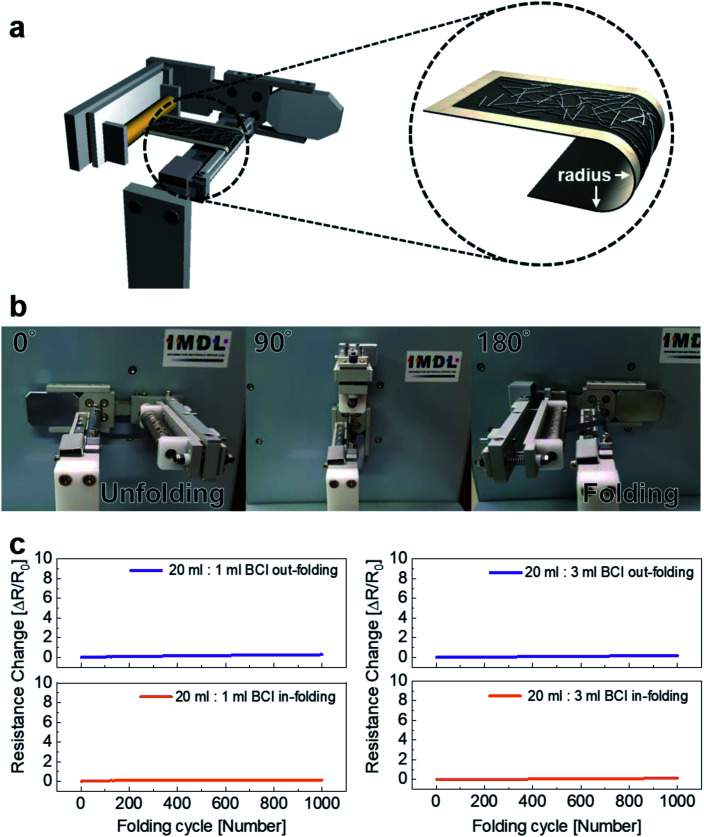
(a) Illustration of a lab-designed folding test system. The enlarged image shows the definition of a folding radius. (b) The picture shows each step of inner folding. (c) Resistance change of the 20 ml : 1 ml BCI and 20 ml : 3 ml BCI paper electrodes measured *in situ* at a fixed outer and inner folding radius of 4 mm for 1000 cycles.

We also conducted a dynamic folding test using a lab-designed folding test system. The apparatus for the folding test is shown in Fig. 6(a). Like the outer and inner bending tests, the folding radius in the in-fold and out-fold tests could be preset, as shown in the enlarged image of Fig. 6(a). Fig. 6(b) shows each step of the out/in dynamic folding tests with changing folding angles and a fixed radius of 4 mm. The initial resistance (*R*_0_) was measured *in situ* when the brush-painted electrode on Hanji was unfolded. After 180° folding, the resistance (*R*) of the brush-painted electrode was measured *in situ* by the folding test system automatically. Like bending fatigue test, the folding test shows excellent flexibility. The inner and outer folding test of 20 ml : 1 ml showed about 0.17 and 0.29 of *R*_change_ and inner and outer folding test of 20 ml : 3 ml showed about 0.18 and 0.13 of *R*_change_, respectively. The lack of change in bending and folding tests indicates that the brush-painted electrode on Hanji can be applied to paper-based flexible electronics as an effective interconnector.

**Fig. 7:**
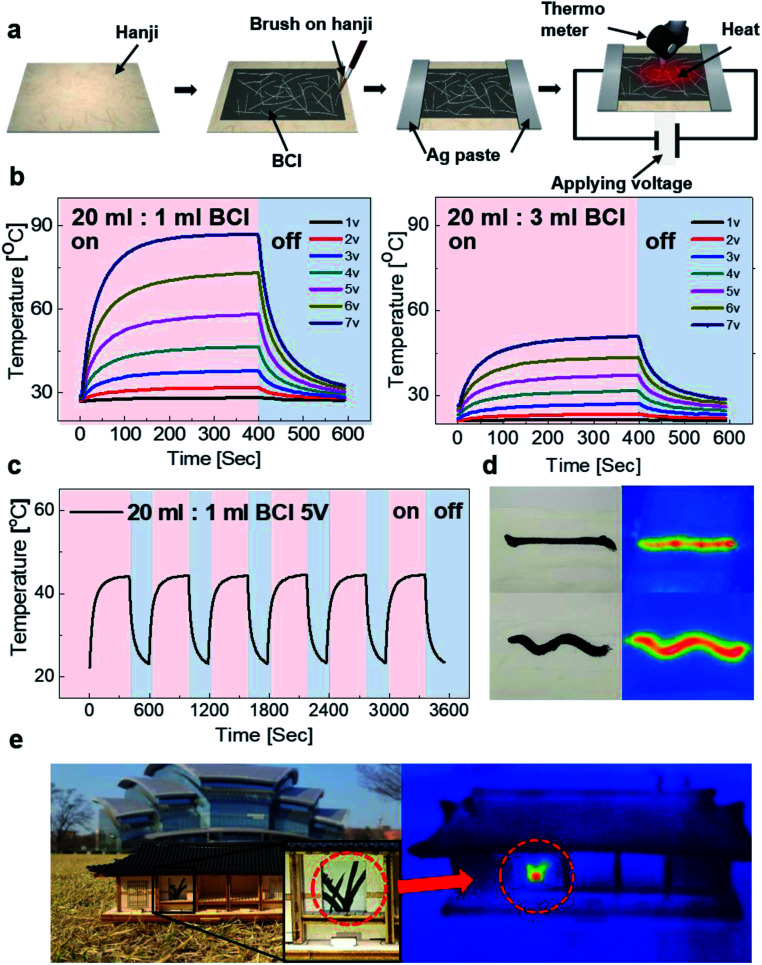
(a) Fabrication process of a flexible Hanji heater with the brush-painted electrode. (b) Temperature profiles of the flexible Hanji heater according to the different DC voltages. (c) Temperature profiles of the brush-painted electrode on Hanji under repeated heating and cooling at 5 V for 1 hour. (d) IR images according to the different shapes of brush painting. (e) Promising application of the flexible Hanji heater with the brush-painted electrode for a traditional Korean architecture called Hanok. The door frame covered by Hanji with the brush-painted electrode showed high temperature to heat up the inner space of Hanok.

To demonstrate the various potentials of the brush-painted electrode on Hanji, we fabricated a Hanji-based paper heater. Fig. 7(a) shows the fabrication process of a flexible Hanji heater. In the process of fabricating the Hanji heater, a three-times brush-painted electrode was fabricated by using a BCI with different mixing ratios of Ag NW suspension and PEDOT:PSS solution (20 ml : 1 ml and 20 ml : 3 ml). The three-times brush-painted electrode on the Hanji showed a sheet resistance of 40 and 87 Ohm per square, respectively. Then we attached contact electrodes (Ag paste and Cu tape) at the edge of the Hanji heater to supply DC power. Fig. 7(b) compares the temperature profiles of the Hanji heater made of BCI-coated electrodes with the different mixing ratios. When DC input voltage was supplied to the Hanji heater through the edge copper (Cu) electrodes, the temperature of the Hanji heater gradually increased to a maximum saturation temperature. As the input voltage increased, the maximum saturation temperature of the Hanji heater increased. Therefore, the temperature of the Hanji heater could be controlled by the input DC voltage. The maximum saturation temperature of each Hanji heater is illustrated below, as determined by eqn (2) and (3) that are induced from joule heat.^36–39^2
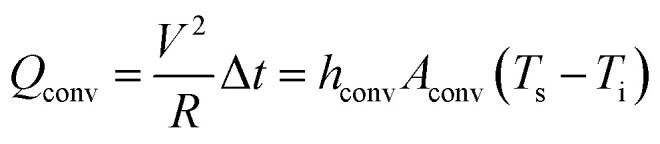
3
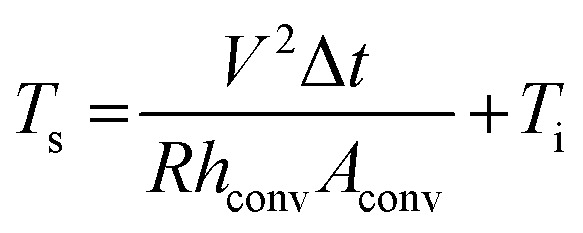


According to thin film heater theory, air convection is one of the main factors of heat dissipation (*Q*_conv_). The *Q*_conv_ is the multiplication of power and operating times. The *Q*_conv_ is also expressed by multiplying the convective heat transfer coefficient (*h*_conv_), surface area (*A*_conv_), the remainder of saturation temperature (*T*_s_) and initial temperature (*T*_i_). According to the preceding eqn (3), the lower resistance and the higher application of voltage marks a high saturation temperature. Therefore, the Hanji heater with the brush-painted electrode (BCI 20 ml : 1 ml) is expected to yield a higher maximum saturation temperature (85.5 °C at 7 V) due to its lower sheet resistance, compared to the BCI 20 ml : 3 ml version (50.7 °C at 7 V). To investigate the durability of the Hanji heater with the brush-painted electrode (BCI 20 ml : 1 ml), we performed repeated heating–cooling tests for one hour. Fig. 7(c) shows the temperature profiles of the Hanji heater according to the repeated heating and cooling tests at 5 V. The Hanji heater rapidly reached a maximum saturation temperature of 40 °C and the maximum saturation temperature did not decrease after six heating–cooling cycles. For one hour heating, the saturation temperature was maintained constant at 40 °C. Even when using Hanji paper, we can increase the temperature to around 40 °C simply by using BCI brush painting, as shown in Fig. 7(c). Considering the simplicity of the process of brush painting on Hanji, any forms, pictures or characters can be used as a conductive electrode for heating. Fig. 7(d) shows the performativity of the straight and curvy lines on Hanji and of IR images, both of which can used for heating. Therefore, we can make a Hanji heater by using a simple drawing of various lines, shapes, forms, or characters. Fig. 7(e) demonstrates a promising application of the Hanji heater to a housing system. In general, the wooden door frames or wallpapers of traditional Korea architecture (Hanok) are covered up with Hanji that maintains the indoor temperature and ventilates the room. The application of the Hanji heater to the wooden door frame covers or wallpapers will enable effective temperature control of the Hanok. The ability of a simple picture or a character drawn on Hanji to act as a heater will facilitate numerous applications of traditional Hanji to modern industry, including housing and architecture.

**Fig. 8:**
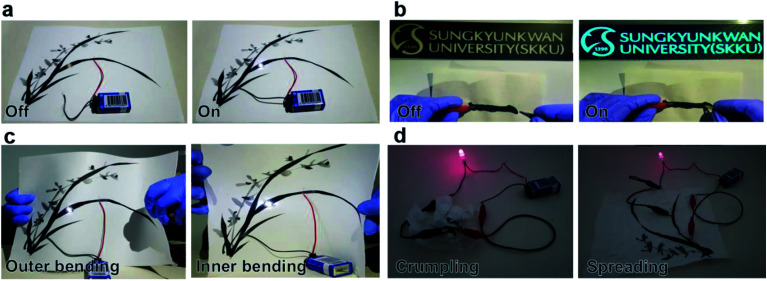
Promising application of the brush-painted electrode as a flexible interconnector. (a) An oriental orchid painting drawn in BCI on Hanji functions as an interconnector. The LED attached on the orchid was illuminated when the battery was connected to the picture. (b) A Chinese character meaning “one” functions as an interconnector for EL. (c) Working of the LED on brush-painted orchid electrode when the Hanji is bent to the outer/inner. (d) The brush-painted electrode can turn on a red LED even when the Hanji is crumpled and spread.

Fig. 8 (a) and (b) show the image of oriental orchid and a Chinese character meaning number 1 that were prepared as a basic design for BCI brush painting. The LED attached on the orchid was successfully turned on when connected to the battery. The LED lighting on the brush-painted picture indicates that the simple drawing of orchid could perform as a flexible interconnector. Furthermore, the commercial EL device connected with the Chinese character was turned on due to the high conductivity of the brush-painted electrodes. The lighting of the LED and EL connected with the brush-painted electrode on Hanji indicates that any picture or characters painted on the Hanji can act as an effective interconnector for optoelectronic devices. In general, Hanji experiences various types of stress due to its nature. Even if Hanji experiences extreme stresses, it can be an effective interconnector for optoelectronic devices due to its outstanding flexibility, which was demonstrated in the results of the bending and folding tests. Fig. 8(c) shows a picture of the orchid painted on a flexible Hanji paper, which functions even at outer/inner bending. The LED on the curved orchid picture on Hanji was still turned on since the cellulose fiber structure and Ag NW network are extremely flexible even under tensile and compressive stresses. Fig. 8(d) shows a picture of the orchid on Hanji when the Hanji paper was crumpled and spread. Even after crumpling and spreading the painting, the orchid picture continued to act as an interconnector for lightening the red LED.

**Fig. 9:**
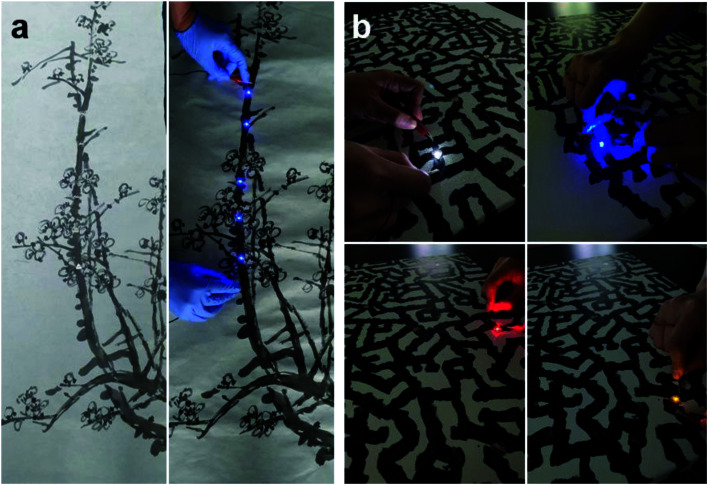
(a) Brush painting of a plum tree on a Hanji substrate, where blue LEDs are embedded in the form of a serial connection. Once DC power was applied to the LEDS attached onto the stem of the plum tree, all of the LEDs were turned on simultaneously. (b) Brush painting of abstract lines on a canvas. The black lines drawn in BCI act as an interconnector for illuminating the LEDs.

Fig. 9(a) shows the serial operation of multi-LEDs attached onto an oriental brush painting on Hanji. Due to the low sheet resistance (11.7 Ohm per square at six brush strokes) of the brush-painted picture, the serially connected blue LEDs were turned on simultaneously when DC power was applied to the painting. The BCI could also be applied on the textile surface of a canvas for western-style painting, as shown in Fig. 9(b). The random chain-like black lines were drawn with a paintbrush in the BCI (20 ml : 1 ml) solution. Several LEDs with different colors were attached onto the black lines of the painting. When we applied DC power to each LED embedded on the brush-painted lines, all the LEDs were successfully turned on. This demonstrated that BCI could be applied in different types of substrate, including paper and textile, and that BCI paintings, either oriental or western-style, could act as electrodes and interconnectors.

## 4 Conclusions

Paintable and writable BCI was fabricated by mixing Ag NW suspension, PEDOT:PSS solution and traditional Korean black ink called “meok”. To use brush-painted pictures or characters as interconnectors and heaters, we optimized the process of brush painting on the traditional Korean paper named “Hanji”. The sheet resistance of the brush-painted BCI on the Hanji substrate decreased with increasing number of brush strokes. At the sixth brush stroke, the brush-painted electrode on Hanji showed a low sheet resistance 11.7 Ohm per square, which is an acceptable value for fabricating interconnectors and heaters, and outstanding mechanical flexibility. During the outer/inner bending and folding tests, there was no change in resistance. During the dynamic fatigue tests, the brush-painted electrode on Hanji showed better durability due to the flexible fiber structure of Hanji and the flexibility of the Ag NW network covering the fiber. Among the diverse applications of the brush-painted picture on Hanji, we suggested a Hanji heater and a flexible interconnector picture made of Hanji. The Hanji heater showed outstanding stability for repeated heating–cooling systems, as demonstrated by its high saturation temperature of 85.5 °C, even at an input voltage of 7 V, due to the low resistance of the brush-painted electrodes. Furthermore, the brush-painted electrodes on Hanji, which contain visual forms, lines, and characters, could act as flexible interconnectors for optoelectronic devices. The high performativity of the brush-painted electrodes will support their utilization as Hanji heaters and flexible interconnectors. In conclusion, the paintable and writable electrode using BCI on Hanji is an effective means to advance the technology of paper-based flexible electrodes.

## Conflicts of interest

The authors declare no competing financial and nonfinancial interests.

## Supplementary Material

RA-010-D0RA04412A-s001
