# Supplementary material for: Paintable and writable electrodes using black conductive ink on traditional Korean paper (Hanji)
Source: RSC Adv. 2020 Jun 29;10(41):24631–41. doi: 10.1039/d0ra04412a (PMC9055205; doi:10.1039/d0ra04412a)
Supplement: RA-010-D0RA04412A-s001 [file RA-010-D0RA04412A-s001.pdf]

Electronic Supplementary Material (ESI) for RSC Advances.
This journal is © The Royal Society of Chemistry 2020

Supporting Information

Paintable and Writable Electrodes Using Black Conductive Ink on Traditional Korean Paper (Hanji)

Yong Jun Kim[a], Sunyoung Yoon[a], Yong-Hwan Cho[a], Gyewon Kim[b], and Han-Ki Kim[a]*

[a]School of Advanced Materials Science and Engineering, Sungkyunkwan University, Suwon, Gyeonggi-do 16419, Korea
[b]Department of Fine Arts, Sungkyunkwan University, 25-2, Sungkyunkwan-ro, Jongno-gu, Seoul, Republic of Korea

E-mail: hankikim@skku.edu Fax: +82-31-201-2462; Tel: +82-31-205-2462

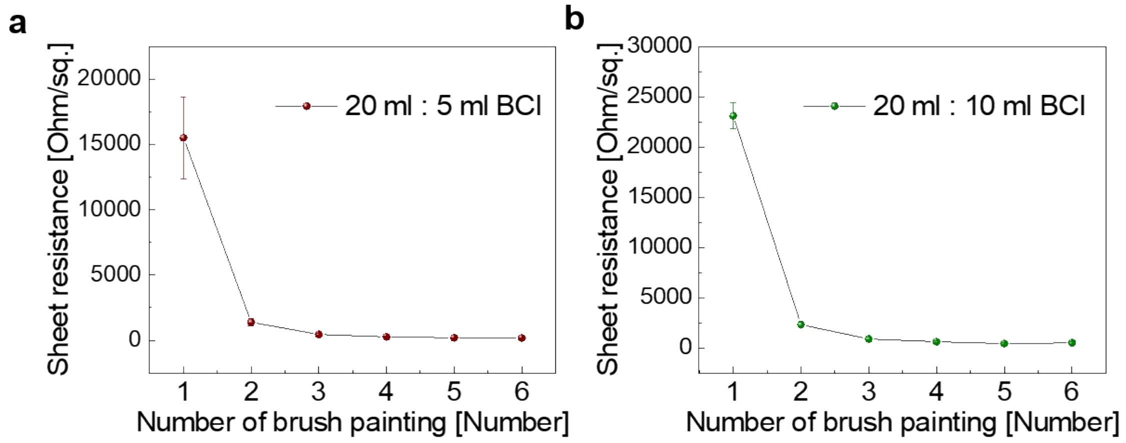

Figure S1. The sheet resistance change of brush painted electrode on Hanji using BCI with Ag NW suspension and PEDOT:PSS solution mixing ratio of (a) 20 ml : 5 ml and (b) 20 ml : 10 ml with increasing number of brush stroke.

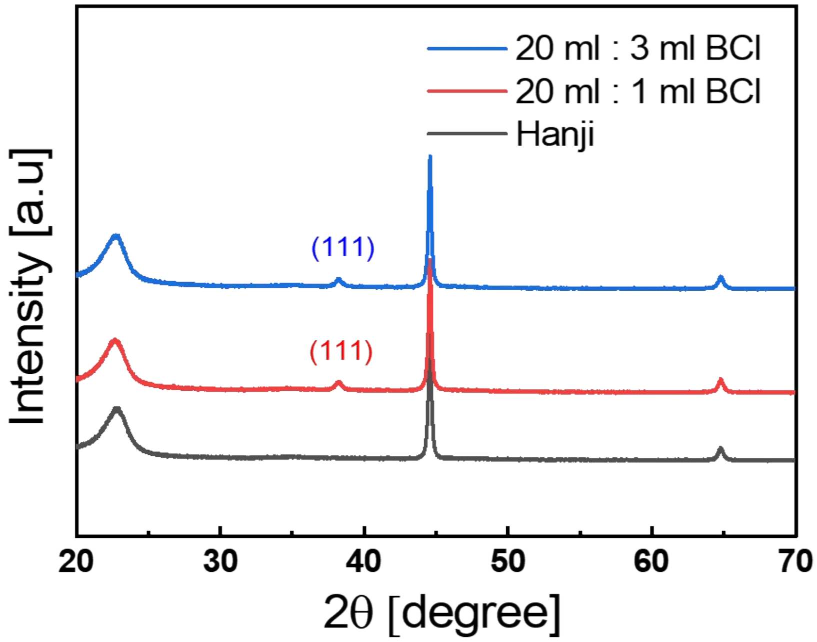

Figure S2. XRD plots of pristine-Hanji, brush painted electrodes on Hanji using BCI with Ag NW suspension and PEDOT:PSS solution mixing ratio of 20 ml : 1 ml and 20 ml : 3 ml.

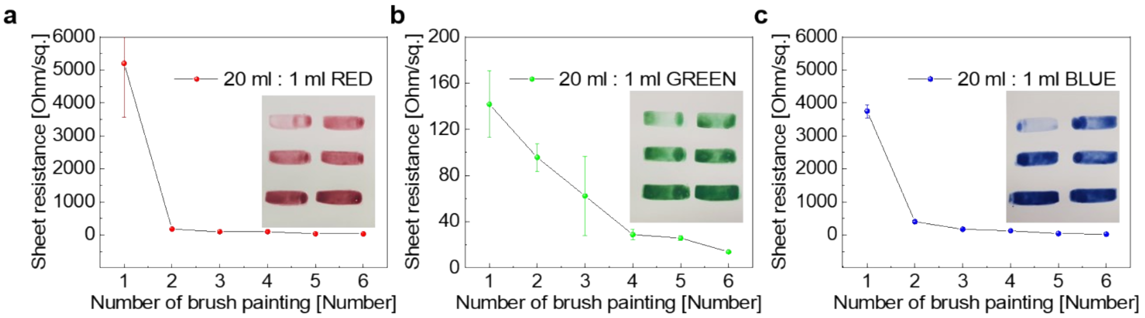

Figure S3. The resistance of brush painted electrode on typical sketch book using conductive ink (pre-BCI 20 ml : 1 ml) mixed with (a) red, (b) green, and (c) blue dye with increasing number of brush stroke. Inset shows the chromaticity change with increasing number of brush stroke.

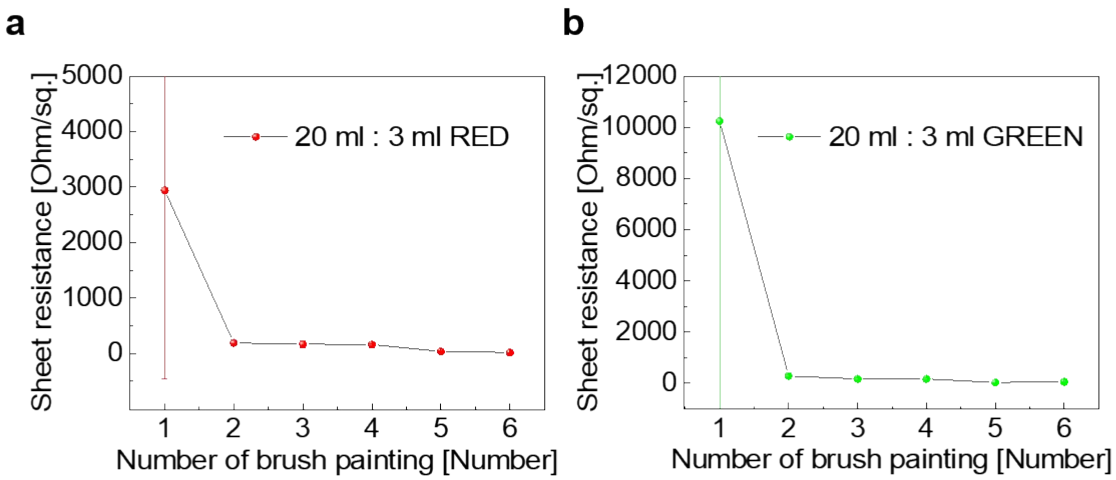

Figure S4. The resistance of brush painted electrode on typical sketch book using conductive ink (pre-BCI 20 ml : 3 ml) mixed with (a) red, and (b) green dye with increasing number of brush stroke.
